# Evaluation of the Diagnostic Performance of Magnetic Resonance Spectroscopy in Brain Tumors: A Systematic Review and Meta-Analysis

**DOI:** 10.1371/journal.pone.0112577

**Published:** 2014-11-13

**Authors:** Wenzhi Wang, Yumin Hu, Peiou Lu, Yingci Li, Yunfu Chen, Mohan Tian, Lijuan Yu

**Affiliations:** Center of PET/CT-MRI, Cancer Hospital of Harbin Medical University, Harbin, 150081, China; Instituto de Investigación Sanitaria INCLIVA, Spain

## Abstract

**Object:**

The aim of this study was to determine the suitability of magnetic resonance spectroscopy (MRS) for screening brain tumors, based on a systematic review and meta-analysis of published data on the diagnostic performance of MRS.

**Methods:**

The PubMed and PHMC databases were systematically searched for relevant studies up to December 2013. The sensitivities and specificities of MRS in individual studies were calculated and the pooled diagnostic accuracies, with 95% confidence intervals (CI), were assessed under a fixed-effects model.

**Results:**

Twenty-four studies were included, comprising a total of 1013 participants. Overall, no heterogeneity of diagnostic effects was observed between studies. The pooled sensitivity and specificity of MRS were 80.05% (95% CI = 75.97%–83.59%) and 78.46% (95% CI: 73.40%–82.78%), respectively. The area under the summary receiver operating characteristic curve was 0.78. Stratified meta analysis showed higher sensitivity and specificity in child than adult. CSI had higher sensitivity and SV had higher specificity. Higher sensitivity and specificity were obtained in short TE value.

**Conclusion:**

Although the qualities of the studies included in the meta-analysis were moderate, current evidence suggests that MRS may be a valuable adjunct to magnetic resonance imaging for diagnosing brain tumors, but requires selection of suitable technique and TE value.

## Introduction

The early detection of brain tumors is associated with significant clinical benefits, but presents a diagnostic challenge. A total of 57,100 new cases of brain tumors were diagnosed in Europe in 2012, and 45,000 deaths were attributed to brain tumors, half of which were glioblastomas [1]. Information on histological grade and tissue diagnosis are important for the clinical management of brain cancers, and are closely related to survival probability. However, there are two major limitations to the histopathological grading of brain tumors, especially gliomas. Firstly, although stereotactic biopsy can adequately represent pathological grading of the whole tumor, potential sampling error of biopsy was inherent. Secondly, it is very difficult to accurate assess residual tumor tissue after cytoreductive surgery [2]. Contrast-enhanced magnetic resonance imaging (MRI) is the current gold standard for guiding neurosurgeons when obtaining biopsy tissue for the diagnosis of brain tumors. However, the results of this technique can sometimes be ambiguous, and differentiating progressive or recurrent brain tumors from radiation-induced injury is difficult using MRI [3]. Proton magnetic resonance spectroscopy (MRS) provides important metabolic information of tumours, such as N-acetyl-aspartate (NAA), choline (Cho), creatine (Cr) at different MRS echo times (TEs), and showed a major advantage without electromagnetic radiation exposure as an imaging technique for guiding brain tumor biopsy procedures [4].

Several recent studies have reported the utility of MRS for brain tumor assessment, with the ability to differentiate between high-grade and low-grade gliomas [5], and between neoplastic and non-neoplastic brain lesions [6]. However, it is difficult todraw conclusions based on individual studies because variations instudy qualities, and different inpatient populations and study designs may cause heterogeneity amongstudy results. To overcome the shortcomings of individual studies, we performed a systematic review and meta-analysis of published data on the diagnostic performanceof MRS for detecting, differentiating, and grading brain tumors, especially gliomas, to determine the diagnostic value of MRS.

## Materials and Methods

### Data sources and search strategy

Electronic searches of the Medline (using PubMed as the search engine) and ProQuest Health & Medical Complete databases were conducted using the terms ‘magnetic resonance spectroscopy’, ‘brain tumor or gliomas’ and ‘sensitivity and specificity’ to identify appropriate studies published in English prior to December 30, 2013. Included studies must have used MRS to detect the occurrence, grade, recurrence, or transformation of brain tumors.

### Study selection

Two authors independently screened the search results by title and abstract. They obtained the full text of each manuscript and excluded studies with overlapping data and studies that did not provide both sensitivity and specificity information for MRS evaluation of brain tumors. Author names, institutions, publication dates, tumor and assessment types were collected for all studies. All the studies were evaluated independently and discussed by the authors until a consensus was reached.

### Data extraction and quality assessment

Two authors independently extracted the data from each study, including information on the first author, year of publication, country, sample size, tumor and assessment type, and sensitivity and specificity of MRS for brain tumors, as well as the risk of bias according to pre-specified criteria from the Cochrane Collaboration’s tool for assessing risk of bias [7]. The following risk-of-bias items were evaluated independently by two authors using standardized methods: sequencing generation, allocation concealment, blinding of patients and study personnel, blinding of outcome assessment, incomplete outcome data, selective reporting, and other biases.

### Data synthesis and statistical analysis

In order to evaluate the diagnostic accuracy of MRS for brain tumors, we calculated the sensitivity, specificity, positive likelihood ratio (PLR), negative likelihood ratio (NLR), diagnostic odds ratio (DOR) and 95% confidence intervals (CI). The result of pathologic tissue diagnosis was the reference standard in all cases. Due to the different diagnostic purpose in multiple studies, different positive sets were defined. For tumor recurrence studies, recurrence was considered as positive and postoperative necrosis was negative. For tumor grading studies, high-grade gliomas (III–IV grade) were positive and low-grade gliomas (I–II) were negative. Statistical heterogeneities in summary effects of PLR, NLR, and DOR were tested in all data using Cochran’s Q test, which approximately follows a χ^2^ distribution with k−1 degrees of freedom (where k is the number of studies included) [8]. The statistic I^2^ = ((Q−(k−1))/Q)×100% was also assessed. I^2^ ranged from 0–100%, with 0–25%, 25–50%, 50–75%, and 75–100% indicating low, moderate, high, and very high degrees of heterogeneity, respectively [9]. We considered a p value <0.05 to indicate significant heterogeneity. Values of diagnostic effects were evaluated usinga fixed-effects or random-effects model, depending on the p value of the heterogeneity test. A summary receiver operating characteristic (SROC) curve was generated based on the sensitivity and specificity of each study for assessing the diagnostic accuracy. Linear regression of the logits of the sensitivity (Se) and specificity (Sp) was used to fit the SROC curve, through the equation D = a+b×S, where D = logit(Se)−logit(1−Sp) = log(OR) and S = logit(Se)+logit(1−Sp), a is the intercept and b is the regression coefficient estimated in the regression equation. D represents the diagnostic log-odds ratio that relates to the test’s diagnostic accuracy for discriminating between disease-positivity and negativity, depending on the threshold used. S represents the threshold for classifying a test as positive. The closer b is to 0, the more evidence exists for a lack of significant heterogeneity with respect to OR. If b differs from 0, the OR is dependent on the threshold used. The SROC curve can be fit weighted by the inverse of the variance of the logarithm of OR from the individual studies corresponding to the area under the SROC curve (AUC). Based on the SROC, when Se equals Sp, where Se = exp(a/2)/[1+exp(a/2)] and 1−Sp = 1/[1+exp(a/2)], Q* = Se = 1−Sp was estimated to represent the diagnostic threshold at which the probability of a correct diagnosis was constant for all subjects. Funnel plot analyses and Egg’s test were used to evaluate publication bias. All statistical tests were performed using mada package in R [10].

## Results

### Study characteristics

**Figure 1:**
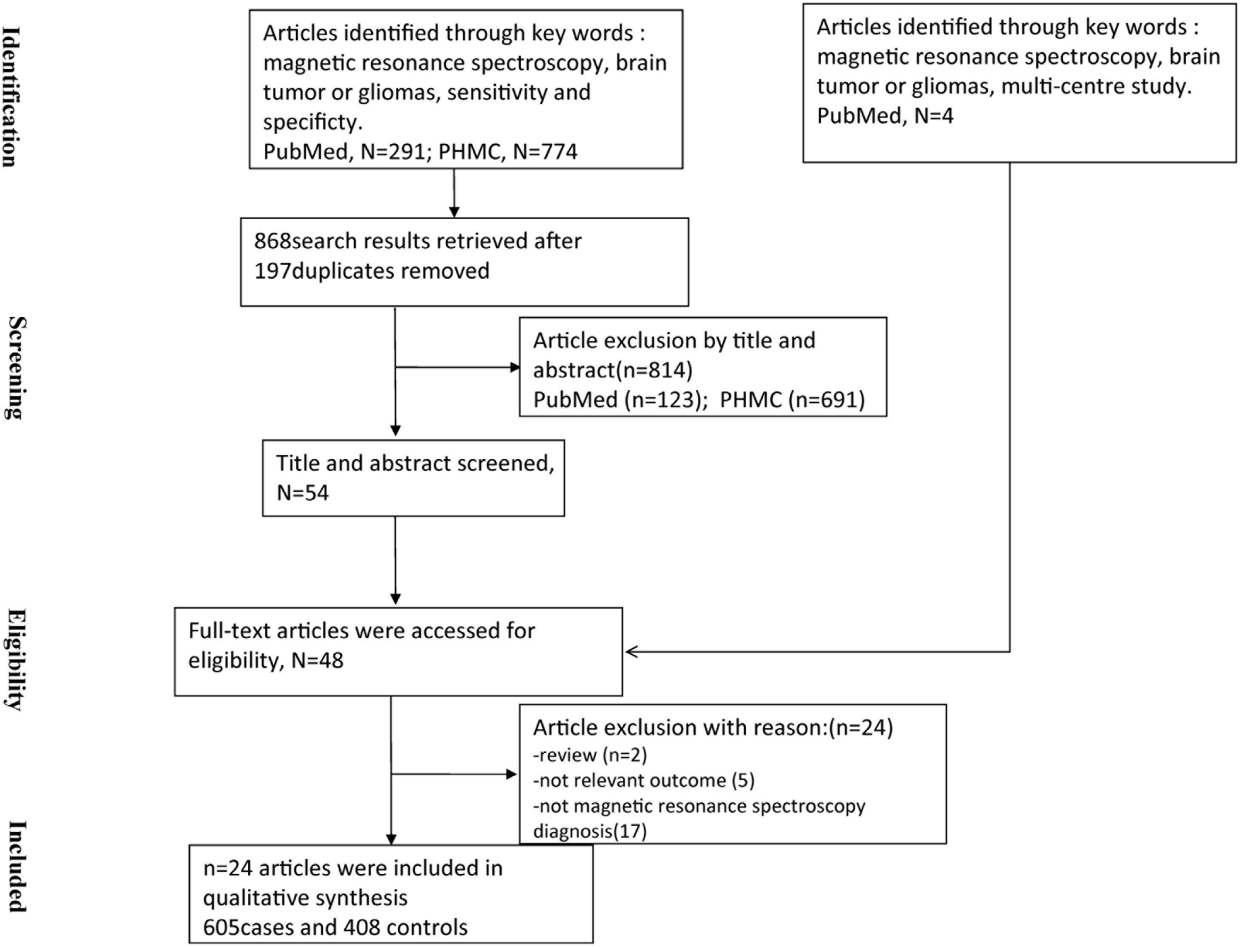
Flow chart showing the process of studies retrieved.

A total of 54 studies were identified after filtering titles and abstracts, and four multi-centre studies including pattern recognition studies was retrieved from PubMed. Finally, full texts of 48 studies were obtained. 24 studies were excluded based on the inclusion criteria, including two studies that were reviews, five studies that did not report the sensitivity and specificity of MRS for brain tumor diagnosis, and seventeen studies that did not use MRS to assess the tumor. The systematic literature search yielded 24 studies including 1013 participants (605 cases and 408 controls, Figure 1). The studies originated from 10 countries or regions (including the USA, Turkey, China, Japan, Norway, Spain, France, Germany, Italy and Egypt) and were published between 1995 and 2013. The sample sizes of the included studies ranged from 12–160 (mean 40).

All the included studies evaluated the diagnostic accuracy of MRS for the detection or grading of brain tumors. Twenty-two studies assessed gliomas [2], [11], [12], [13], [14], [15], [16], [17], [18], [19], [20], [21], [22], [23], [24], [25], [26], [27], [28], [29], [30], [31], two study assessed ependymomas and primitive neuroectodermal tumors [32], [33]. Seven studies evaluated the diagnostic power of recurrence [11], [14], [15], [19], [22], [26], [27], nine studies evaluated the grade [2], [13], [17], [20], [21], [23], [24], [28], [31], five studies evaluated the detection [16], [18], [29], [32], [33], one evaluated residual tumor [12], and two evaluated tumor metastases [25], [30]. The detailed diagnostic power are shown in Table 1.

**Table 1:** Characteristics of all included studies.

Study	Center	Period	Cancer	Type	TP	FP	FN	TN	Technique	Method(ms)	Cutoff
Reddy et al (2013)	Single	Adult	Gliomas	Recurrent	2	1	2	7	–	–	–
Pamir et al (2013)	Single	Adult	Gliomas	Residual	12	0	2	6	SV	LTE = 135	Cho/Cr ↑ 20%
Sahin et al (2013)	Single	Adult	Gliomas	Grade	6	4	0	10	CSI	STE = 30	Cho/Cr = 1.3
Seeger et al (2013)	Single	Adult	Gliomas	Recurrent	16	4	7	13	CSI	LTE = 135	Cho/Cr = 2.33
Amin et al (2012)	Single	Adult	Gliomas	Recurrent	11	0	7	6	SV	STE = 30	Cho/Cr = 1.5
Crisi et al (2013)	Single	Adult	Gliomas	Detection	18	3	5	15	SV	STE = 35	–
Liu et al (2012)	Single	Adult	Gliomas	Grade	19	1	3	9	SV	LTE = 144	Cho/Cr = 2.01
Liu et al (2012)	Single	Adult	Gliomas	Grade	16	1	6	9	SV	LTE = 144	Cho/NAA = 2.49
Liu et al (2012)	Single	Adult	Gliomas	Grade	17	3	5	7	SV	LTE = 144	NAA/Cr = 0.97
Peng et al (2012)	Single	Adult	Gliomas	Detection	19	6	3	13	CSI	LTE = 144	Cho/Cr = 3.16
Peng et al (2012)	Single	Adult	Gliomas	Detection	18	2	4	17	CSI	LTE = 144	Cho/NAA = 2.13
Peng et al (2012)	Single	Adult	Gliomas	Detection	14	2	8	17	CSI	LTE = 144	Cho/Cho-n = 1.28
Guillevin et al (2011)	Single	Adult	Gliomas	Recurrent	5	0	3	13	SV	TE = 35/144	(Cho/NAA-Cho/Cr)/(Cho/NAA) = 0.046
Zou et al (2011)	Single	Adult	Gliomas	Grade	15	0	3	12	CSI	LTE = 135	NAA/Cho = 0.265, ADC = 1118.1×10^−6 ^mm^2^/s
Server et al (2011)	Single	Adult	Gliomas	Grade	54	0	5	15	CSI	LTE = 135	Cho/NAA = 1.78
Prat et al (2010)	Single	Adult	Gliomas	Recurrent	11	1	0	12	–	–	–
Zeng et al (2011)	Single	Adult	Gliomas	Grade	21	2	4	10	CSI	LTE = 144	Cho/Cr = 2.04
Zeng et al (2011)	Single	Adult	Gliomas	Grade	22	4	3	8	CSI	LTE = 144	Cho/NAA = 2.20
Zeng et al (2011)	Single	Adult	Gliomas	Grade	19	4	6	8	CSI	LTE = 144	NAA/Cr = 0.72
Senft et al (2009)	Single	Adult	Gliomas	Grade	28	17	8	10	CSI	LTE = 144	Cho_mean_ = 1.51
Senft et al (2009)	Single	Adult	Gliomas	Grade	31	6	5	21	CSI	LTE = 144	Cho_max_ = 2.02
Senft et al (2009)	Single	Adult	Gliomas	Grade	26	14	10	13	CSI	LTE = 144	CE
Senft et al (2009)	Single	Adult	Gliomas	Grade	28	9	8	18	CSI	LTE = 144	Cho/Cr = 0.58
Hlaihel et al (2009)	Single	Adult	Gliomas	Metastases	4	1	1	15	SV/CSI	LTE/STE = 32/136	Cho/Cr = 2.4
Hlaihel et al (2009)	Single	Adult	Gliomas	Metastases	5	8	0	8	SV/CSI	LTE/STE = 32/136	Cho/Cr = 1.7
Hlaihel et al (2009)	Single	Adult	Gliomas	Metastases	2	5	3	11	SV/CSI	LTE/STE = 32/136	rCBV = 2
Hlaihel et al (2009)	Single	Adult	Gliomas	Metastases	2	7	3	9	SV/CSI	LTE/STE = 32/136	rCBV = 1.75
Hlaihel et al (2009)	Single	Adult	Gliomas	Metastases	4	7	1	9	SV/CSI	LTE/STE = 32/136	rCBV = 1.5
Zeng et al (2007)	Single	Adult	Gliomas	Recurrent	18	0	1	9	CSI	LTE = 144	Cho/Cr = 1.71
Palumbo et al (2006)	Single	Adult	Gliomas	Recurrent	17	1	2	10	SV	LTE = 144	Cho/Cr = 2.0
Fayed et al (2006)	Single	Adult	Gliomas	Grade	17	2	1	10	SV	STE = 30	Cho/Cr = 1.56
Floeth et al (2005)	Single	Adult	Gliomas	Detection	34	3	0	13	SV	LTE = 135	–
Law et al (2003)	Single	Adult/Child	Gliomas	Grade	117	6	3	44	CSI	STE = 6	Cho/Cr = 1.08
Wang et al (1995)	Single	Child	Astrocytoma	Detection	10	1	1	14	SV	LTE = 135/270	–
Wang et al (1995)	Single	Child	Ependymoma	Detection	3	2	1	20	SV	LTE = 135/270	–
Wang et al (1995)	Single	Child	Neuroectodermal tumor	Detection	9	1	2	14	SV	LTE = 135/270	–
Vellido et al (2012)	Multiple	Adult	Gliomas	Metastases	7	3	3	27	SV	STE/LTE = 20/135	–
Vellido et al (2012)	Multiple	Adult	Gliomas	Metastases	9	5	1	25	SV	STE/LTE = 20/135	–
Vellido et al (2012)	Multiple	Adult	Gliomas	Metastases	6	2	4	28	SV	STE/LTE = 20/135	–
Vellido et al (2012)	Multiple	Adult	Gliomas	Metastases	9	5	1	25	SV	STE/LTE = 20/135	–
Tate et al (2006)	Multiple	Adult	Gliomas	Grade	44	2	5	12	SV	STE = 20	–
Davies et al (2008)	Single	Child	Astrocytoma	Detection	16	0	0	18	SV	STE = 30	–

### Exploration of heterogeneity and sensitivity analysis

**Figure 2:**
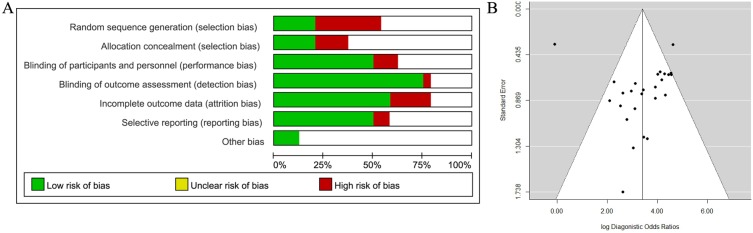
Methodological quality and publish bias assessment. (A) Risk of bias graph. The items of bias were independently evaluated by two authors. If the study reported all of the sensitivities and specificities of genes which were measured DNA methylation status, selective reporting was defined as low risk. (B) Funnel plot to assess bias in estimates of diagnostic odds ratio caused by small-study effects.

We assessed the risk of bias for each study, and the detailed standard and results for each item of bias are shown in Table S1 and Figure S1. The risk of bias is summarized in Figure 2A. In general, the risk of bias was low or unclear in most studies for many assessed items. Six studies stated that the sequences of participants were generated randomly and were therefore defined as low risk. The sponsors of 30%–67% of studies had authorship and were not involved in data collection, assessment of tumors, or interpretation of the outcomes. The sensitivities and specificities of all the different diagnostic methods were reported in 50% of studies, indicating no selective reporting. Three studies were reported to be free of other sources of bias.

In order to evaluate the heterogeneity of the diagnostic effects of MRS, we performed heterogeneity tests for PLR, NLR, and DOR (Table 2). No significant heterogeneity of diagnostic effects was observed (p>0.05, I^2^ = 0%), as described in Table 2. We therefore adopted a fixed-effects model for all measures in the meta-analysis.

**Table 2:** The heterogeneity analysis of diagnostic effects.

	Estimate [95% CI]	Log(Estimate) [95% CI]	df	Q	P-value	I^2^
PLR	3.53 [2.71–4.60]	1.28 [1.05–1.52]	41	29.77	0.90	0%
NLR	0.29 [0.24–0.36]	−1.31 [−1.53–1.09]	41	41.03	0.47	0.062%
DOR	14.66 [9.81–21.92]	2.86 [2.42–3.30]	41	41.22	0.46	0.54%

PLR: positive likelihood ratio. NLR: negative likelihood ratio. DOR: diagonistics odd ratio. Estimate [95% CI]: the pooled effect measure with the corresponding 95% confidence interval. Log(Estimate) [95% CI]: logarithmic transformation of the pooled effect measure with the corresponding 95% confidence interval. df: degrees of freedom. Q and P-value were the Q value and p value of Cochran’s Q test.

Funnel plots were used to demonstrate the effects of small study size for each diagnostic imaging modality, to assess publication bias by examining the relationship between the effect measure (log DOR) and its standard error. As shown in Figure 2B, relatively symmetrical funnel plots suggested potential publication bias in five of the 24 studies, which fell outside the funnel. Publication bias was evaluated using Egg’s test, which found no significant differences (p = 0.40). This suggests that there was no trend towards higher levels of test accuracy among studies with smaller sample sizes.

Meta-regression analysis was used to assess factors affecting the diagnostic accuracy of MRS. We suspected that different tumor types, diagnostic purposes, patient period, technique of MRS and TE could affect the sensitivity and false positive rate of tumor diagnosis. We therefore used true and false positive rates as responses and studied whether the above five factors can affect the diagnostic accuracy through meta-regression analyses, respectively. As shown in Table 3, the p values for the tumor type, diagnostic purpose, MRS technique and TE in the fixed-effects model were not significant for true positive rate or false positive rate. However, periods of patient had significant effects on sensitivity and false positive rate of MRS (p value<0.001 respectively). In addition, differential of tumor grad had significant correlation with false positive rate of MRS (p value = 0.01). We therefore concluded that the diagnostic accuracy of MRS was robust for different types, MRS technique and TE in brain tumors.

**Table 3:** Meta-regression of potential risk of bias of methodological characteristics affecting the diagnostic sensitivity of MRS.

Factor	Label	Sensitivity		False positive rate	
		Coefficient	P value	Coefficient	P value
Cancer	Gliomas	−1.04	0.22	1.57	0.11
	Ependymoma	−1.54	0.26	0.59	0.68
	Neuroectodermal tumor	−1.06	0.38	0.43	0.78
Diagnose	Grade	0.22	0.49	1.05	0.01
	Metastases	−0.58	0.16	0.62	0.17
	Recurrent	−0.38	0.35	−0.17	0.77
	Residual	0.22	0.80	−0.80	0.63
Period	Adult	2.23	<0.001	3.04	<0.001
	Child	0.34	0.48	−1.20	0.03
Technique	SV	0.43	0.62	0.03	0.97
	CSI	0.56	0.51	1.22	0.12
TE	STE	1.19	0.20	0.26	0.81
	LTE	0.73	0.38	0.14	0.87

### Meta-analysis and diagnostic accuracy

**Figure 3:**
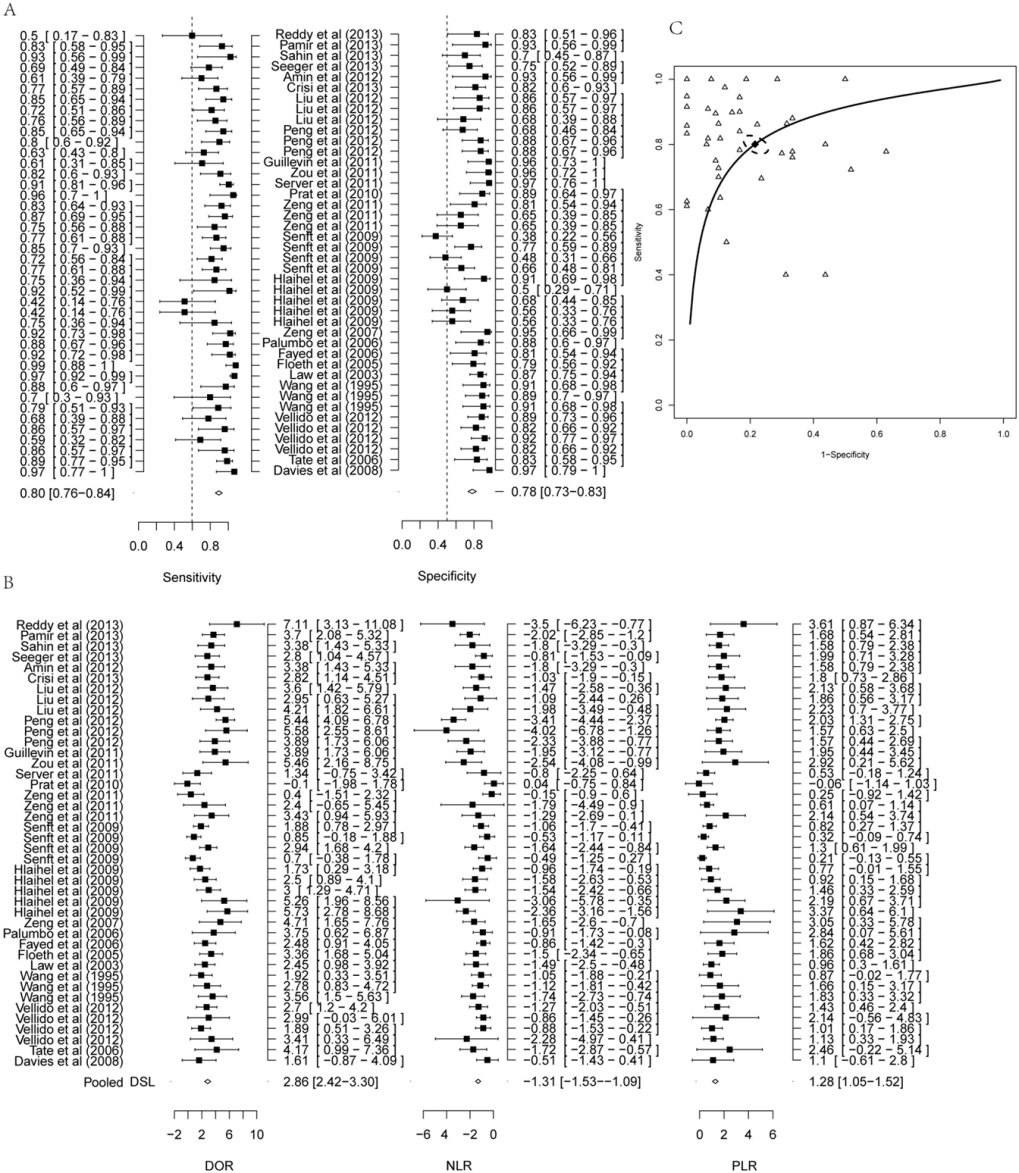
Forest plot of estimate of diagnostic accuracy of MRS. (A) Forest plot of estimate of sensitivity and specificity of MRS. (B) Forest plot estimate of PLR, NLR and DOR of MRS. (C) SROC curve of diagnostic performance of MRS from all studies. Solid line represents the ROC curve, and dotted line represented 95% confidence ellipse. Hollow triangle represented observed data from each study and solid rhombus represented the summary estimate.

Meta-analysis revealed that the overall sensitivity and specificity of MRS were 80.05% (95% CI: 75.97–83.59%) and 78.46% (95% CI: 73.40%–82.78%, Figure 3A), respectively. The overall PLR after logarithmic transformation was 1.28 (95% CI: 1.05–1.52) corresponding to 3.53 (95% CI: 2.71–4.60, Table 2 and Figure 3B). The NLR after logarithmic transformation was −1.31 (95% CI: −1.53 to −1.09) corresponding to 0.29 (95% CI: 0.24–0.36, Table 2 and Figure 3B). The DOR after logarithmic transformation was 2.86 (95% CI: 2.42–3.30) corresponding to 14.66 (95% CI: 9.81–21.92, Table 2 and Figure 3B). In general, MRS thus demonstrated high diagnostic accuracy.

We generated an SROC curve based on the sensitivity and specificity of each study. The regression coefficient b was 0.002 (95% CI: −0.37–0.37), where b was close to 0 indicating a lack of heterogeneity, which was consistent with the results of heterogeneity analysis of diagnostic effects. The AUC showed relatively high diagnostic accuracy (Figure 3C, AUC = 0.78). Based on the SROC curve, the Q* metric was calculated as 84.22% (95% CI: 80.69%–87.21%), when the sensitivity equaled the specificity. These results suggest that MRS can be used for screening brain tumors with good diagnostic accuracy.

### Stratified meta analysis

**Figure 4:**
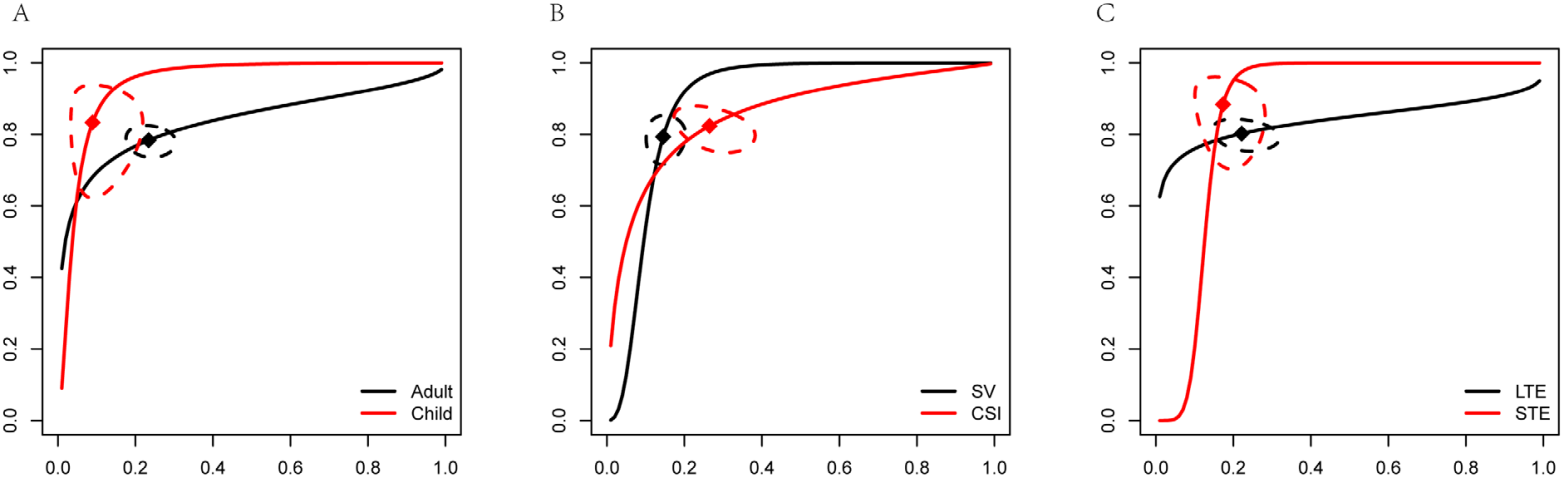
SROC curve of diagnostic performance of stratified meta-analysis. (A) Adult and child. (B) SV and CSI. (C) LTE and STE.

**Figure 5:**
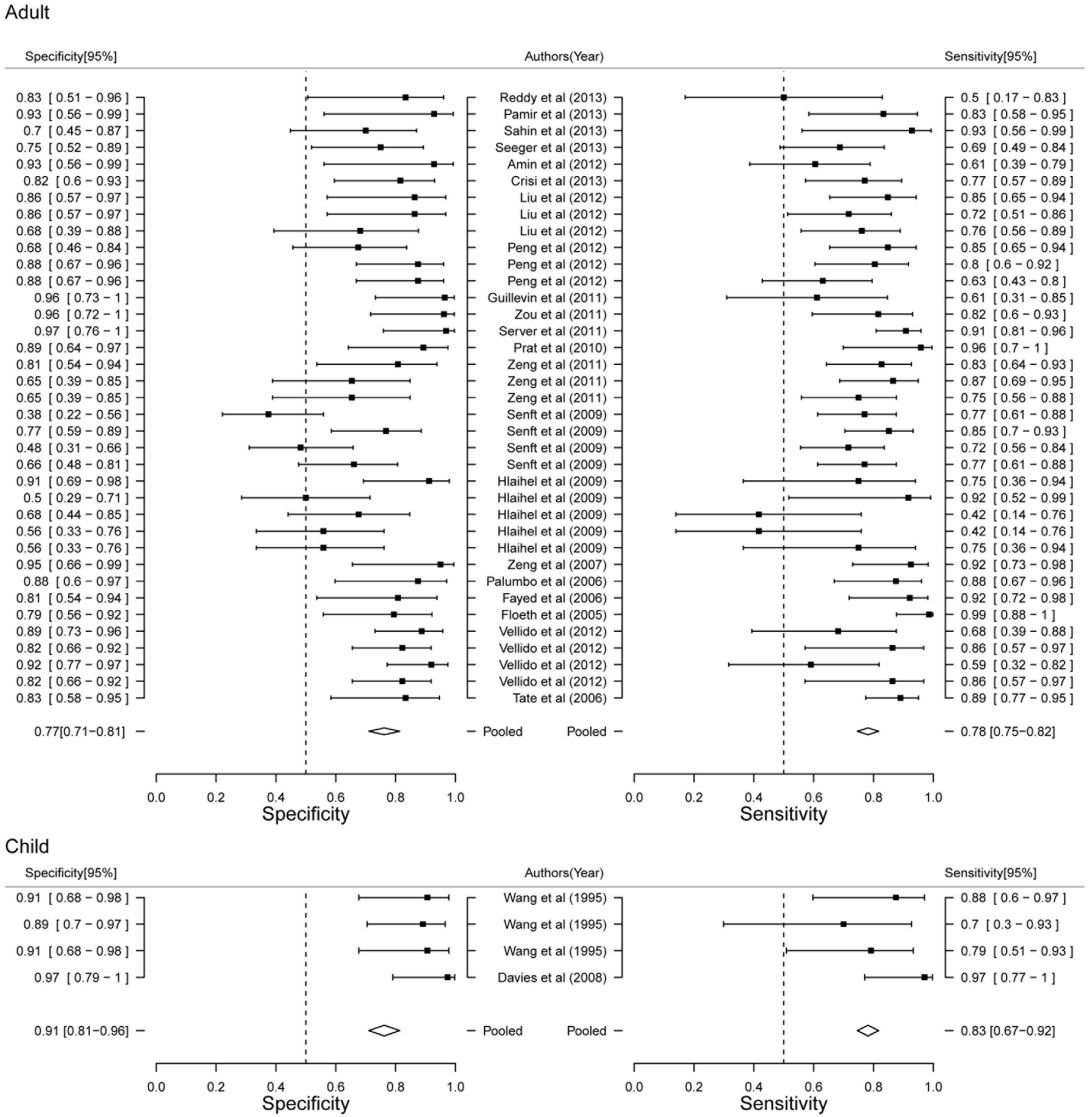
Forest plot of estimate of diagnostic accuracy of adult and child stratified meta-analysis.

**Figure 6:**
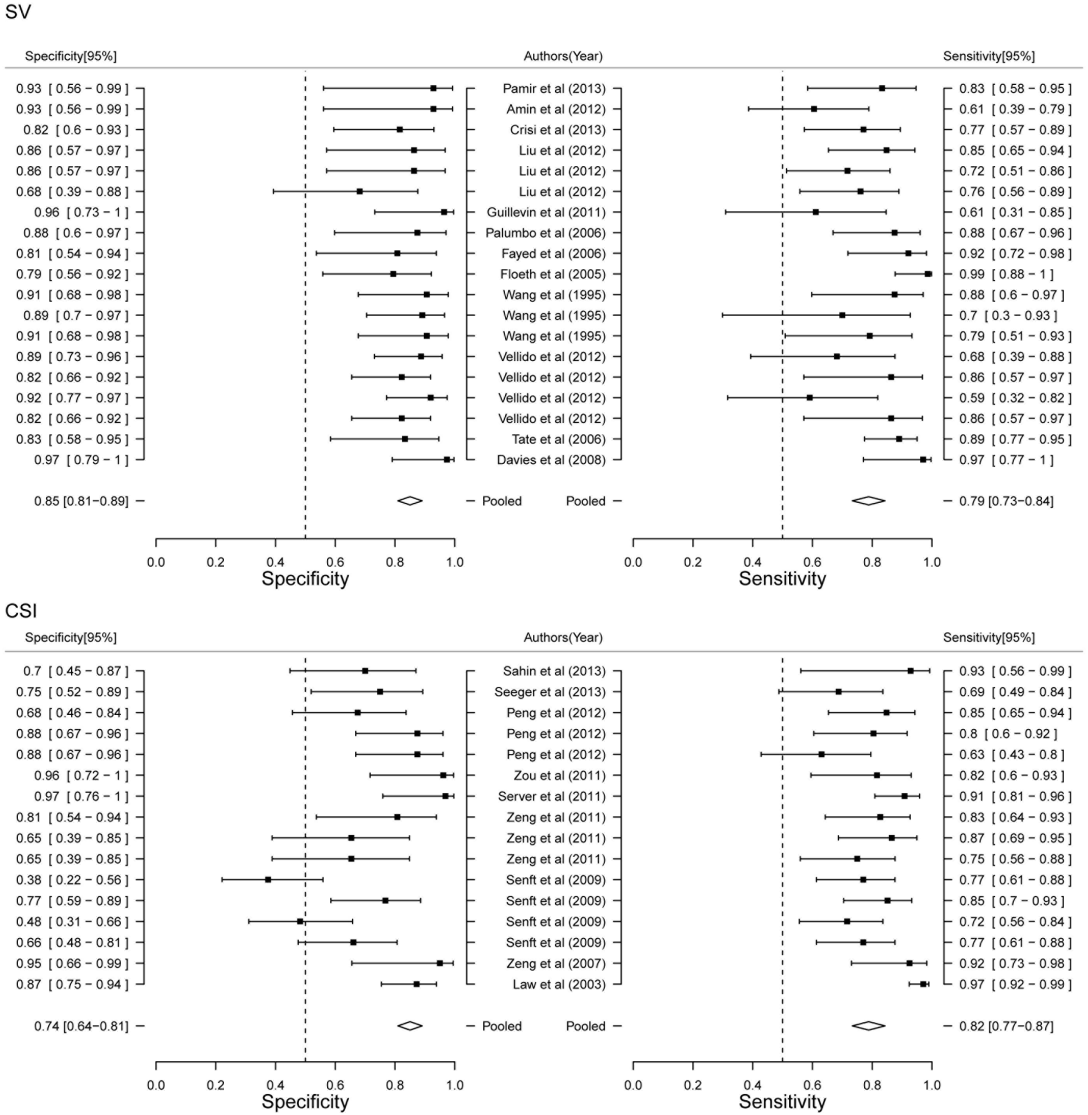
Forest plot of estimate of diagnostic accuracy of SV and CSI stratified meta-analysis.

**Figure 7:**
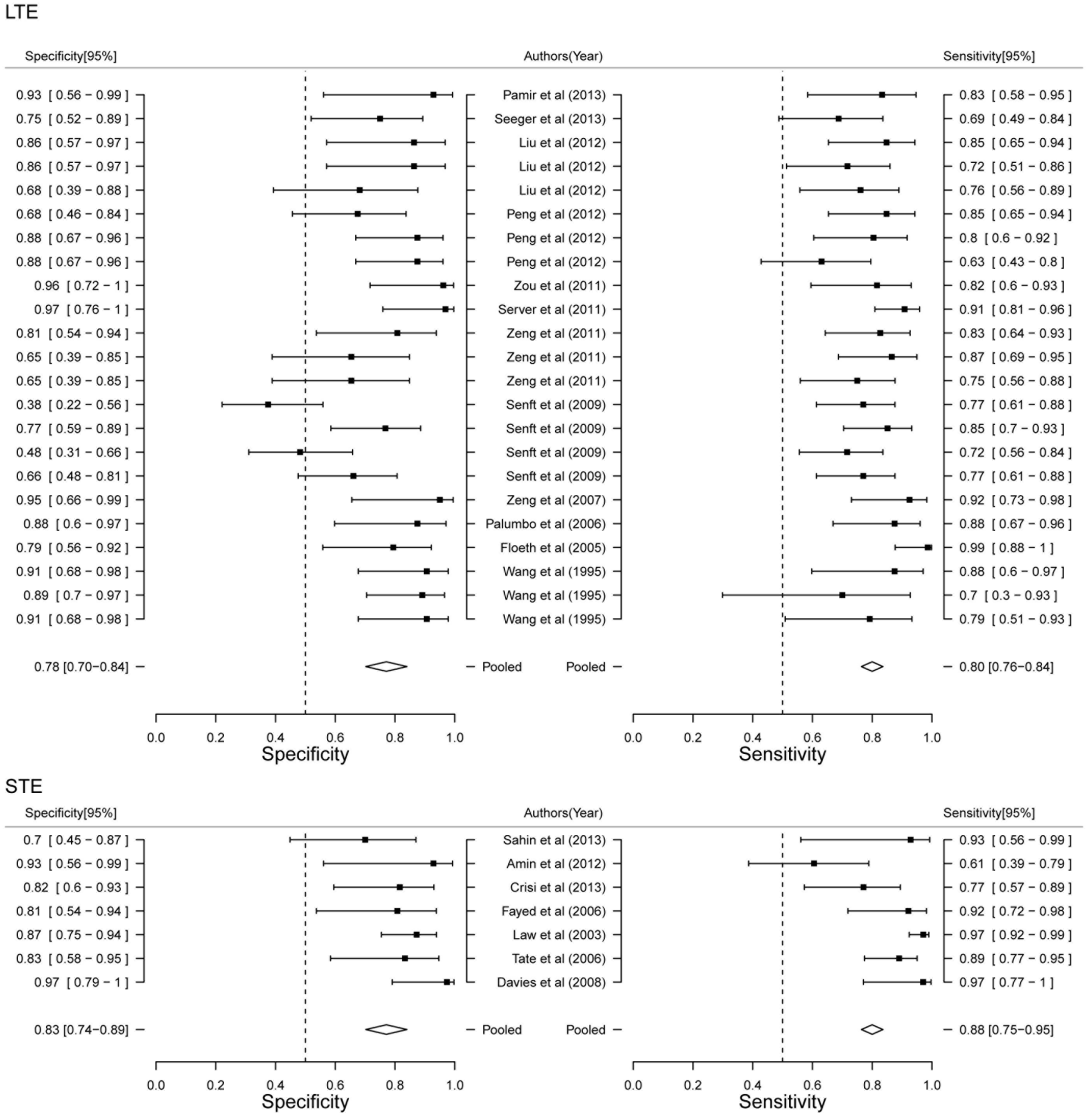
Forest plot of estimate of diagnostic accuracy of LTE and STE stratified meta-analysis.

In order to further detailed analyze the diagnostic power of MRS, we performed Stratified meat analysis based on the period of patients, MRS technique and TE value. Diagnostic power of MRS between adult and child showed that child had more high accuracy than adult (AUC 0.89 VS. 0.77, Figure 4A). Diagnostic performance of MRS showed both higher sensitivity (83.37% VS. 78.38%) and specificity (91.06% VS. 76.60%) in child (Figure 5). Our results limited the very few studies on child, that will be more accurate with the increase of the number of studies. Although AUC value of SV was higher than CSI (0.89 VS. 0.79, Figure 4B), CSI had higher sensitivity (82.39% VS. 79.35%) and SV had higher specificity (85.49% VS. 73.52%, Figure 6). Two techniques of MRS has its own advantage. Finally, we analyzed the diagnostic power of LTE and STE. STE showed slightly higher AUC (0.79 VS. 0.73, Figure 4C), and had higher sensitivity (88.40% VS. 80.23%) and specificity (77.86% VS. 73.52%, Figure 7). Although some studies adopted double standard including both LTE and STE, diagnostic power has not been improved (sensitivity = 80.05% [95% CI: 75.97%–83.59%] and specificity 78.46% [95% CI: 73.40%–82.78%], respectively).

## Discussion

Contrast-enhanced structural MRI is the method of choice for diagnosing brain tumors, especially follow-up of brain metastasis. However, the differentiation of locally-recurrent brain metastasis in many patients is difficult using contrast-enhanced structural MRI [34]. Various imaging techniques such as positron-emission-tomography (PET), single-photon emission computed tomography (SPECT), MRS and perfusion-weighted MRI (PWI) have been used to differentiate tumors. PET has been used to diagnose brain metastases [35], but it limits to small lesion size [36], long time interval between PET scans [37] and requiring of ionising radiation source [4]. Although SPECT provided higher sensitivity (90%) and specificity (92%) than PET, the major disadvantage of SPECT over PET was lower spatial resolution [38]. PWI and MRS as advanced MRI techniques can be successfully used to differentiate brain tumors. PWI provided high sensitivity (70%–100%) and specificity (95%–100%) [37]. MRS even reached sensitivity and specificity of 100% [39]. However, these studies investigating advanced MRI techniques have mostly been based on limited numbers of patients. In addition, small size of the lesion, or susceptibility artifacts near to the lesion may negatively affect the analysis and interpretation of MRS data [40], [41], thus limiting its diagnostic accuracy. It is difficult to draw conclusions about the diagnostic accuracy of MRS for brain tumors based on individual studies, and pooled studies thus represent a useful approach for assessing its diagnostic performance.

The present systematic review and meta-analysis included 24 studies, comprising a total of 1013 participants, with 605 cases and 408 controls. Overall, the methodological quality of the included studies was moderate, with no heterogeneity or publication bias, despite the fact that the different studies used different criteria for positivity. Meta-analytically, MRS demonstrated slightly high sensitivity and specificity for discriminating brain tumors (pooled estimates of 80.58% and 78.46%, respectively), suggesting that it is a suitable and accurate diagnostic technique for brain tumors. Based on stratified meta analysis, MRS showed higher sensitivity and specificity in STE than LTE. CSI had higher sensitivity and SV had higher specificity. Diagnostic accuracy of MRS between adult and child need to increase the number of studies on child.

The present meta-analysis had several limitations. First, no large-scale prospective validation studies have been carried out by stereotactic biopsy. Second, the included studies did not provide sufficient information to assess the diagnostic values of other imaging techniques for comparison with multimodal imaging studies. Third, the included studies used a combination of different controls (normal, necrosis, and low-grade, respectively) as reference standards for determining diagnostic accuracy. Fourth, although we evaluated the diagnostic accuracy of MRS for brain tumors, more gliomas were included.

In conclusion, despite the limitations of this systematic review and meta-analysis, current evidence suggests that MRS may be an appropriate, non-invasive method for diagnosing brain tumors.

## Supporting Information

Figure S1 Risk of bias summary.
(PDF)

Table S1 Detailed of risk of bias table.
(DOC)

Checklist S1 PRISMA Checklist.
(DOC)
