# Supplementary material for: Evaluation of the Diagnostic Performance of Magnetic Resonance Spectroscopy in Brain Tumors: A Systematic Review and Meta-Analysis
Source: PLoS One. 2014 Nov 13;9(11):e112577. doi: 10.1371/journal.pone.0112577 (PMC4231038; doi:10.1371/journal.pone.0112577)
Supplement: Figure S1 — Risk of bias summary. (PDF) [file pone.0112577.s001.pdf]

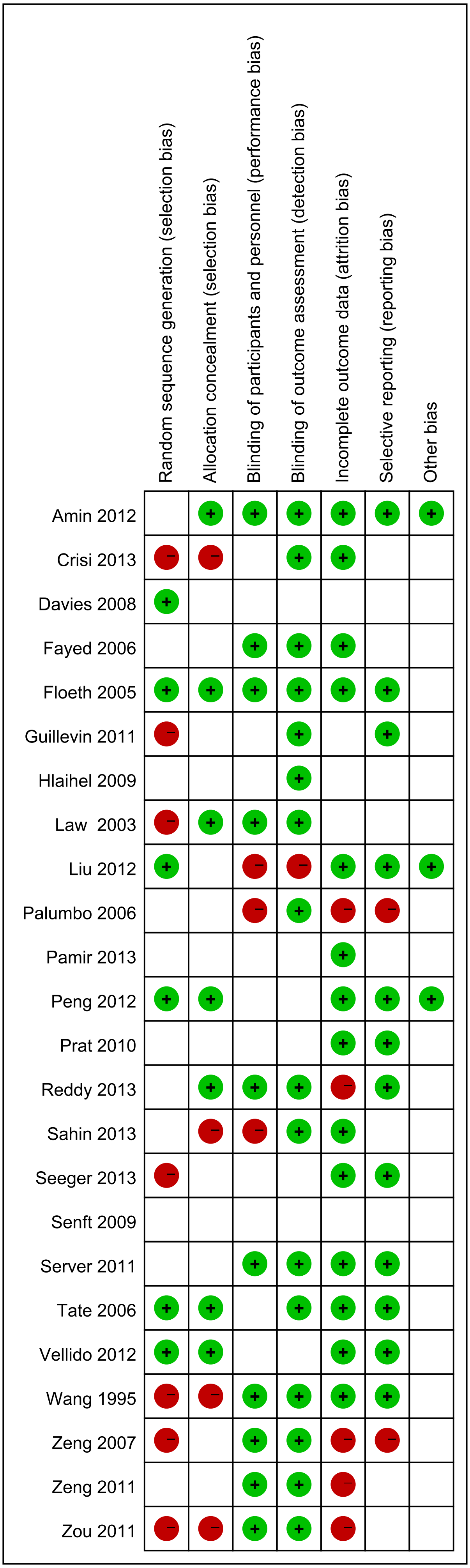
Random sequence generation (selection bias)
Allocation concealment (selection bias)
Blinding of participants and personnel (performance bias)
Blinding of outcome assessment (detection bias)
Incomplete outcome data (attrition bias)
Selective reporting (reporting bias)
Other bias
Amin 2012
Crisi 2013
Davies 2008
Fayed 2006
Floeth 2005
Guillevin 2011
Hlaihel 2009
Law 2003
Liu 2012
Palumbo 2006
Pamir 2013
Peng 2012
Prat 2010
Reddy 2013
Sahin 2013
Seeger 2013
Senft 2009
Server 2011
Tate 2006
Vellido 2012
Wang 1995
Zeng 2007
Zeng 2011
Zou 2011
